# How China is responding to the challenge of myopia

**Published:** 2022-03-01

**Authors:** Mayinuer Yusufu, Ningli Wang

**Affiliations:** 1 PhD Candidate: Centre for Eye Research Australia, Royal Victorian Eye and Ear Hospital, East Melbourne and Department of Surgery (Ophthalmology), University of Melbourne, Melbourne, Australia.; 2 Professor of Ophthalmology: Beijing Institute of Ophthalmology, Beijing Tongren Eye Center, Beijing Tongren Hospital, Capital Medical University and Beijing Ophthalmology and Visual Sciences Key Laboratory, Beijing, China.

**China's coordinated approach to reducing myopia amongst school children has started to reduce the proportion of children affected.**

**Figure:**
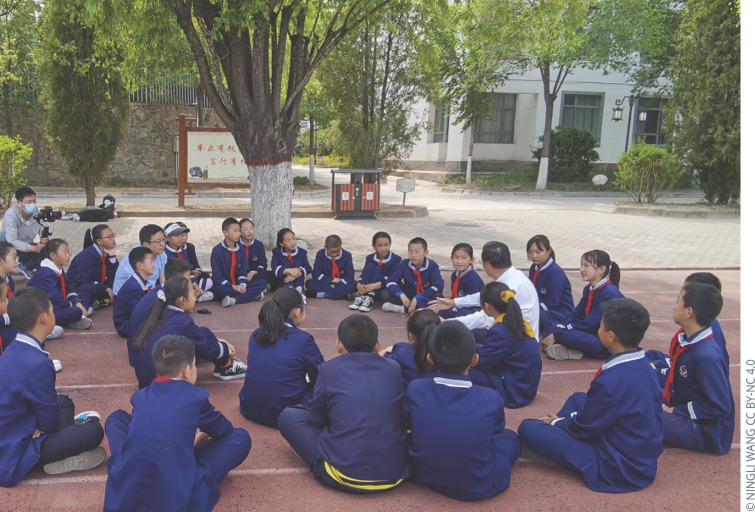
Children learn about eye health at school. **CHINA**

Due to intensified intellectual competition, increased digital screen time, insufficient outdoor exercise, and lack of eye care awareness, the myopia rate is rising rapidly among children and adolescents in China. In 2014, the proportion of elementary, junior, and senior high school students with presenting visual acuity <6/6 reached 45.7%, 74.4%, and 83.3% respectively,^1^ largely due to myopia. The Chinese government has recognised myopia as a major public health problem and showed strong leadership in tackling the issue.

In addition to serving as the focus of National Sight Day for several consecutive years and receiving focused attention from the National Health Commission, myopia control is now at the centre of a national strategy promulgated by the Central Committee of the Communist Party. Led by the Ministry of Education (MOE), eight ministries issued an implementation plan for control of children's myopia. The plan emphasised collective action across society and defined roles for parents, schools, the health sector, students, and the eight government departments. The plan set a target of lowering the rate of children's myopia by 0.5% a year by 2030, and by 1% a year in areas with a high prevalence of myopia.^2^ A new work plan for the period 2021–25 was issued in 2020, involving 15 ministries and taking into account the impact of Covid-related restrictions.^3^

Both plans emphasise that government entities at all levels will be evaluated based on their performance and will be held accountable, thereby ensuring that myopia control as a national strategy gains the attention of the whole of society and the active engagement of all relevant departments. The education sector plays the main role, with strict limitations on homework for younger children, regulations on the use of cellphones and the internet, and a target of 2 hours daily outdoors.

The National Health Commission has issued guidelines for evidence-based techniques for myopia control.^4^ The capacity to provide high-quality spectacles to the children who need them is also being increased, with more universities approved to offer programs in optometric medicine (21 universities), optometry (37 universities) and health management (86 universities).^5^

Students and their parents are being encouraged to focus on lifestyle changes and good eye health habits, as guided by the National Committee for the Prevention of Blindness and relevant expert groups. Proven techniques such as increased outdoor time have been emphasised and misconceptions corrected, such as the idea that wearing glasses can harm children's eyes. Products that claim the ability to reduce myopia have been more tightly regulated, and for-profit cram schools, a major contributor to academic pressure, have been restricted.

In 2019, the overall childhood myopia rate fell to 50.2% (from 53.6% in 2018). However, due to COVID, the rate increased to 52.7% in 2020, still achieving the target of an annual reduction of 0.5% in the first two years of the plan.^6^ Despite the many challenges posed by the current pandemic, and the very significant complexities involved in coordinating the activities of 15 contributing ministries, the success of China's myopia control plan provides an important model for other countries facing climbing rates of childhood myopia due to enhanced access to intensive schooling and other social changes.
